# Helical self-assembly of a mucin segment suggests an evolutionary origin for von Willebrand factor tubules

**DOI:** 10.1073/pnas.2116790119

**Published:** 2022-04-04

**Authors:** Gabriel Javitt, Deborah Fass

**Affiliations:** ^a^Department of Chemical and Structural Biology, Weizmann Institute of Science, Rehovot 7610001, Israel

**Keywords:** mucins, von Willebrand factor, evolution, protein self-assembly, cryo-electron microscopy

## Abstract

The glycoprotein von Willebrand factor (VWF) contributes to hemostasis by stanching injuries in blood vessel walls. A distinctive feature of VWF is its assembly into long, helical tubules in endothelial cells prior to secretion. When VWF is released into the bloodstream, these tubules unfurl to release linear polymers that bind subendothelial collagen at wound sites, recruit platelets, and initiate the clotting cascade. VWF evolved from gel-forming mucins, the polymeric glycoproteins that coat and protect exposed epithelia. Despite the divergent function of VWF in blood vessel repair, sequence conservation and shared domain organization imply that VWF retained key aspects of the mucin bioassembly mechanism. Here, we show using cryo-electron microscopy that the ability to form tubules, a property hitherto thought to have arisen as a VWF adaptation to the vasculature, is a feature of the amino-terminal region of mucin. This segment of the human intestinal gel-forming mucin (MUC2) was found to self-assemble into tubules with a striking resemblance to those of VWF itself. To facilitate a comparison, we determined the residue-resolution structure of tubules formed by the homologous segment of VWF. The structures of the MUC2 and VWF tubules revealed the flexible joints and the intermolecular interactions required for tubule formation. Steric constraints in full-length MUC2 suggest that linear filaments, a previously observed supramolecular assembly form, are more likely than tubules to be the physiological mucin storage intermediate. Nevertheless, MUC2 tubules indicate a possible evolutionary origin for VWF tubules and elucidate design principles present in mucins and VWF.

## Significance

Extracellular proteins with mechanical functions often require specialized assembly processes to form covalent oligomers. Progress in tissue bioengineering and repair will benefit from an understanding of how to harness and manipulate these processes. Here, we show that a particular supramolecular assembly mode was pre-encoded in the ancient domain organization common to gel-forming mucins and von Willebrand factor, glycoproteins that are deceptively different due to their divergence for distinct mechanical tasks. This finding highlights symmetry principles and building blocks retooled in nature to construct polymers with wide-ranging properties. These building blocks and knowledge of their self-assembly can be used to design new polymeric structures.

Many extracellular proteins in higher organisms comprise multiple copies of characteristic structural domains. As these proteins evolved to their current forms, replication of primordial domains increased the physical spans of the proteins and allowed for functional diversification, such as the acquisition of multiple, distinct protein–protein interaction sites (1). Further increasing functional variation, whole gene duplications and exon shuffling gave rise to protein families such as collagens, laminins, and the large set of serine proteases involved in blood clot formation and degradation (2–4). By comparing structures of related modern-day proteins, it may be possible to deduce their ancestral origins and shed light on the fundamental principles by which new activities emerged, or by which new functionalities can be designed.

**Fig. 1.**
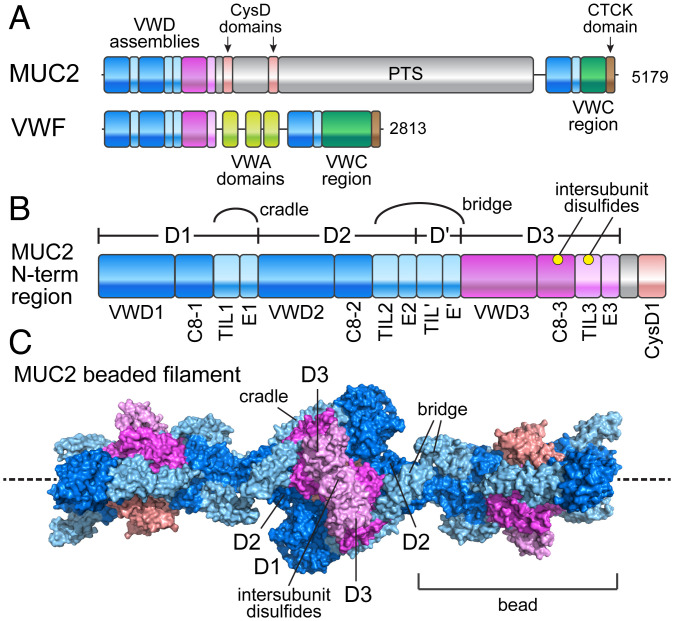
Domain organization of MUC2 and VWF. (*A*) MUC2 and VWF primary structures. Human MUC2 has a PTS stretch of more than 2,300 amino acids with 60% threonine and 20% proline content, whereas VWF has VWA domains in this region. Protein lengths in amino acids are shown to the *Right*. The CTCK domain mediates disulfide-linked dimerization of the carboxy termini (17). (*B*) The map of the MUC2 amino-terminal ∼1,400 amino acids (*N*-term) is expanded to show all domains (50). Cradle refers to a cysteine-rich region that allows the D1 and D2 assemblies to embrace a D3 assembly from another subunit in the filament (22). Bridge refers to a longer cysteine-rich region that extends from the D2 assembly in one bead to the D3 assembly in the neighboring bead. Cysteines forming intersubunit disulfides during mucin assembly are indicated by yellow balls. (*C*) Three beads in the beaded filament of the MUC2 amino-terminal region (22) are shown colored according to panel *B*.

One extracellular protein family that arose through domain replication and gene duplication is the gel-forming mucins, huge glycoproteins that coat and protect the epithelia in the lungs, intestines, and other organs. These mucins have three von Willebrand Factor type D (VWD) assemblies at the amino terminus and often another VWD assembly near the carboxyl terminus, together with von Willebrand Factor type C (VWC) domains and a carboxyl-terminal cystine knot (CTCK) (Fig. 1*A*). The central regions of gel-forming mucins are rich in proline, threonine, and serine (PTS) amino acid residues, providing numerous O-glycosylation sites. Some mucins also contain short, cysteine-rich CysD domains within the PTS region. Phylogenetic studies concluded that the architecture of the mucin amino terminus arose early in Ctenophora (comb jellies). In contrast, CysD domains evolved later, first appearing in Bilateria and found together with the PTS region in Deuterostomia (5, 6).

A remarkable variation on the theme of mucins arose with the evolution of the complex vasculature in the vertebrate lineage (3, 7, 8). von Willebrand factor (VWF), a glycoprotein responsible for hemostasis, has a similar domain organization and is homologous to mucins in its amino- and carboxyl-terminal regions (5, 9, 10). Like mucins, VWF has three VWD assemblies at the amino terminus and one VWD assembly, in addition to VWC and CTCK domains, at the carboxyl terminus (Fig. 1*A*). However, VWF contains three von Willebrand Factor type A (VWA) domains in its central portion instead of the mucin PTS region. The VWA1, VWA2, and VWA3 domains have specific roles in blood clotting or VWF turnover (11–14). The shared domain architecture of the mucin and VWF termini contrasts with the different central regions, indicating that VWF evolved to utilize the capabilities of the ancestral mucin termini (9) while its central part was adapted for its distinct biological niche.

The amino and carboxy termini of mucins and VWF mediate their assembly into higher order structures (15, 16). For both mucins and VWF, the CTCK domains of two polypeptides associate in the endoplasmic reticulum (ER) to generate intermolecularly disulfide-bonded dimers (17, 18). These dimers progress to the Golgi apparatus, which is a more acidic environment than the ER. Under acidic conditions, the amino termini of mucins and VWF form noncovalent intermolecular interactions that juxtapose VWD3 assemblies from different CTCK-linked dimers. The VWD3 assemblies then become disulfide bonded to one another to produce long, disulfide-linked polymers (19–22), although these polymers are thought to remain highly compact until secretion.

Before release from the cell, VWF and mucin polymers are stored in dedicated secretory granules. Mucins are packed in spherical granules about 10 µm in diameter (22) and VWF in cigar-shaped compartments known as Weibel–Palade bodies (WPBs) ∼5 µm long and 0.3 µm wide in endothelial cells (23). Transmission electron microscopy (TEM) of WPBs showed that their distinct shape is due to quasi-crystalline bundles of aligned VWF tubules (21, 24–26). The amino-terminal region of VWF forms the tubular scaffold, while the VWA, VWD4, VWC, and CTCK domains are thought to project outward from the tubule (27, 28). VWF polymers unfurl from the tubules (29) and are secreted into the bloodstream in a latent form until they are activated by high shear at sites of vascular damage (30, 31). In contrast, mucins are secreted from their storage granules onto the epithelial surface, where an increase in pH and a decrease of calcium concentration cause a massive expansion of the glycoproteins and formation of hydrogels (32–36). To our knowledge, there have been no reports of a tubular storage form for mucins prior to secretion.

We recently reported the cryo-electron microscopy (cryo-EM) structure of the amino-terminal region of the intestinal mucin MUC2 (Fig. 1*B*) and showed that it forms a beads-on-a-string filament (Fig. 1*C*). This filament scaffold is proposed to properly position the cysteines in the VWD3 assembly for formation of intermolecular disulfides as required for polymerization (22). The filament with the surrounding PTS regions and carboxy termini in full-length MUC2 likely constitutes the stored form of the glycoprotein at low pH prior to secretion from cells (22). The persistence length of the beaded string appeared to be short relative to the dimensions of the mucin storage granules, consistent with the apparent lack of long-range order in these granules. The different granular storage forms of mucins and VWF may reflect the unique selection pressures on their secretion and function. Evidence to date implied that VWF evolved de novo the capability of assembling a tubular scaffold.

In this work, we show that a mucin segment has an intrinsic ability to form tubules, a property that may have been exploited during the evolution of VWF. We present residue-resolution structures of VWF and MUC2 amino-terminal segment tubules, enabling comparison between them and an analysis of the features that stabilize each one. Moreover, the elements that may suppress tubule formation in the context of full-length mucins are discussed.

## Results

### MUC2 and VWF Form Tubules at Acidic pH.

**Fig. 2.**
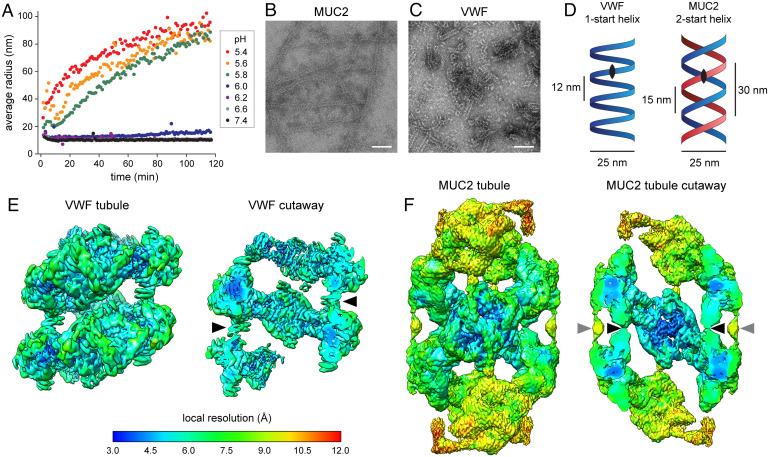
Tubule formation by the amino-terminal region of MUC2. (*A*) DLS of MUC2 D1D2D3 revealed a sharp transition to large species between pH 6.0 and pH 5.8. Radius refers to the size of a sphere with similar diffusion. (*B*) MUC2 D1D2D3 tubules obtained by incubation at pH 5.4 were visualized by negative stain TEM. Scale bars, 100 nm (*B* and *C*). (*C*) Tubules of VWF D1D2 plus D′D3 obtained at pH 6.0 were visualized by negative stain TEM. (*D*) Diagram of one-start and two-start helices, formed by the amino-terminal regions of VWF and MUC2, respectively. The red and blue helices in the two-start helix are identical. The black eye shape indicates the twofold symmetry of each helical strand. The two-start helix has an additional symmetry axis relating the two separate strands (i.e., the red and blue helices) (59). (*E*) Helical reconstruction of VWF tubules. The *Left* shows the map surface from outside the tubule; the *Right* is a cutaway through the center of the tubule. Maps are colored by resolution according to the scale *Below*, which also applies to panel *F*. Arrowheads in the cutaway point to contacts between successive helical turns. (*F*) Helical reconstruction of MUC2 tubules. The *Left* shows the map surface from outside the tubule; the *Right* is a cutaway view. Black arrowheads indicate contacts between helical turns analogous to those seen in VWF; gray arrowheads indicate additional contacts unique to MUC2.

While investigating the mucin polymerization mechanism, we observed that the CysD1 domain was necessary for the MUC2 amino-terminal region to form filaments at a pH value similar to that of the Golgi, i.e., pH 6.2 (22). We subsequently discovered using dynamic light scattering (DLS) that a dimeric MUC2 amino-terminal fragment spanning the D1D2D3 region but lacking CysD1 (Fig. 1*B*) could also self-assemble, but only at a lower pH (Fig. 2*A*). Remarkably, in TEM samples prepared at pH 5.4, the D1D2D3 fragment exhibited tubules (Fig. 2*B*) that resembled the VWF tubules found in WPBs, both with diameters of ∼25 nm (21, 24). To compare the two tubules in more detail, we produced tubules of the VWF amino-terminal segment according to a reported method (21) (Fig. 2*C*). The highest pH that we found to produce well-formed tubules, as evaluated by TEM, was pH 5.4 for MUC2 and pH 6.0 for VWF.

MUC2 and VWF tubules were imaged by cryo-EM, and images of overlapping segments along the tubules were reconstructed into three-dimensional (3D) volumes using a helical reconstruction strategy (*SI Appendix*, Fig. S1). From these initial volumes, it was apparent that the MUC2 and VWF tubules share certain features but also differ in fundamental aspects. Both tubules are composed of beads similar in shape and dimensions to the beads seen in the MUC2 amino-terminal filaments (22) and in a low-resolution reconstruction of VWF tubules (21) (Fig. 1*C*). These beads contain a central disulfide-bonded dimer of two D3 assemblies, with each D3 embraced by the D1D2 portion of a different protein molecule. The VWF and MUC2 tubules also both display twofold symmetry perpendicular to the helix axis. They differ, however, in that the VWF tubule is a one-start helix (21, 27), whereas the MUC2 tubule is a two-start helix (Fig. 2*D*).

Using the initial maps, 3D helical refinement with imposed helical n-start and dyad symmetry was used to obtain refined helical parameters. The VWF tubule has a helical twist of 86.0° (about 4.2 subunits per turn) and an axial rise of 2.85 nm per subunit. These values are in close agreement with previous results, which reported a helical twist of 85.6° and an axial rise of 2.62 nm for the low-resolution reconstruction of the VWF tubular scaffold (21). The MUC2 tubule has a helical twist of ∼83.2° (about 4.3 subunits per turn) and an axial rise of ∼6.95 nm per subunit. Due to the larger axial rise of the two-start compared to the one-start helix, a helical turn of MUC2 (its lead) extends almost 30 nm along the helix axis, whereas a turn of VWF extends only about 12 nm (Fig. 2*D*). The pitch of the MUC2 helix, namely, 15 nm, is slightly longer than the 12-nm pitch of VWF (Fig. 2*D*). The MUC2 and VWF tubule maps indicated contacts between one helical layer and the next, which in MUC2 are made between the two separate, intertwined helices (Fig. 2 *E* and *F*). The MUC2 helix map showed additional interturn contacts not seen in VWF (Fig. 2*F*).

**Fig. 3.**
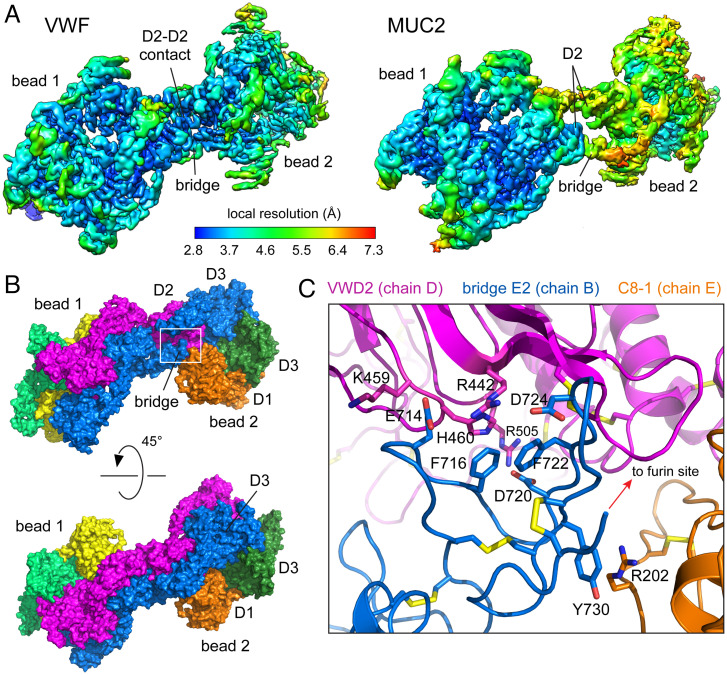
Interactions between beads along the tubule helix. (*A*) Two-bead maps of VWF and MUC2 were colored and filtered according to local resolution estimate, as indicated on the scale *Below*. D2 assemblies from neighboring beads make intimate contacts in the VWF tubule. In addition, the bridge following D2 of one bead is closely packed against D2 of the neighboring bead in VWF but not in MUC2. (*B*) The molecular model of two VWF beads colored by protein chain highlights the ternary interaction between a bridge arising from bead 1 (blue), a D2 assembly in bead 2 (magenta), and the D1 assembly of the other chain in bead 2 (orange). (*C*) Details of the ternary interaction. Amino acids participating in cation–π interactions and salt bridges at the interface are labeled. The furin cleavage site (RSKR, residues 760 to 763) and the surrounding polypeptide were not resolved.

Although useful for calculating helical properties, the helical refinements produced reconstructions of only moderate resolution, probably due to the flexibility of the tubules. To obtain higher resolution maps, local alignments and reconstructions were calculated for tubule subregions (Fig. 3*A* and *SI Appendix*, Table S1 and Fig. S2). The VWF subregion map contained two adjacent beads along the path of the helix at an overall nominal resolution of 3.4 Å, and both beads were of similar resolution. The MUC2 subregion map also consisted of two adjacent beads at a nominal resolution of 3.4 Å, but one bead appeared at higher resolution than the second. The resolution difference between the beads in the MUC2 map may indicate greater flexibility between beads compared to the VWF tubule. An atomic model was built into the VWF map (*SI Appendix*, Fig. S3), aided by the VWF D′D3 crystal structure (PDB ID 6N29) (37), enabling a residue resolution analysis of the contacts involved in VWF tubule formation. Domains of MUC2 obtained from the filament structure (Protein Data Bank identifier [PDB ID] 6TM2) and a crystal structure of the D3 assembly (PDB ID 6RBF) were manually placed into the MUC2 map, providing insight into differences compared to VWF and to the MUC2 filament structure (22).

### Bridging Successive Beads in VWF and MUC2.

The connections between successive beads in the tubules described here and in the MUC2 filament (22) were made by a “bridge” consisting of the TIL2-E2-TIL′-E′ region (Fig. 1*B*). Even in the highest resolution two-bead maps obtainable, the end of E2 and the beginning of TIL′ could not be traced. In VWF, a furin cleavage site is present in the E2-TIL′ junction, and proteolysis occurs intracellularly during VWF bioassembly (26, 31, 38). Due to furin cleavage during recombinant protein production, we assembled the VWF tubules from the D3 dimer and D1D2 fragments purified separately (21). Density maps showed that the region around the furin cleavage site was exposed and flexible. The recombinant MUC2 D1D2D3 segment used for tubule assembly in this study was intact. Nevertheless, the MUC2 E2-TIL′ junction was also unresolved, suggesting that it is a flexible joint. Of note, MUC2 has been reported to be cleaved near this junction after secretion in vivo (39, 40).

Despite the shared flexibility of part of the bridge in VWF and MUC2, the bridge plays different roles in the stabilization of the two tubules. Aside from the furin cleavage region, the remainder of the VWF bridge is highly ordered. The E2 module of a VWF protein molecule arising from one bead makes numerous contacts with the D1 and D2 assemblies from different protein molecules in the neighboring bead (Fig. 3*B*). This ternary interaction may stabilize both the bead assembly and the relative orientation of beads. The contact between the bridge and D1 includes a cation–π interaction between Y730 and R202, while the contact between the bridge and D2 includes cation–π interactions between F716 and H460 and between F722 and R442 (Fig. 3*C*). In the same region, a row of salt bridges is made by the intercalation of residues E714 and D724 from the bridge between R442 and K459 in D2, and another salt bridge was found between Asp720 and Arg505 (Fig. 3*C*). Further supporting the interaction between beads in VWF, direct D2–D2 contacts are made at the interface between beads (Fig. 3 *A*, *Left*). In contrast, apposing MUC2 D2 domains appear only very weakly associated in the tubule (Fig. 3 *A*, *Right*). Additionally, interactions are not evident in the MUC2 tubule between the bridge and D1 or D2. For comparison, extensive D2–D2 interactions were noted between MUC2 beads in their filamentous form, and the bridge appeared to buttress this contact (22). The observation that an intact MUC2 bridge accommodates filament formation when CysD1 is present and tubule formation at lower pH when CysD1 is absent indicates that the bridge is flexible (Movie S1), consistent with the low local resolution of this region in both the tubule (Fig. 3*A*) and filament forms (22). The MUC2 tubule thus appears to be stabilized mainly by quaternary interactions between turns of the helix, whereas the more rigid packing of the bridge in VWF appears to dictate the geometry of its tubule.

### VWF and MUC2 Tubule Models.

**Fig. 4.**
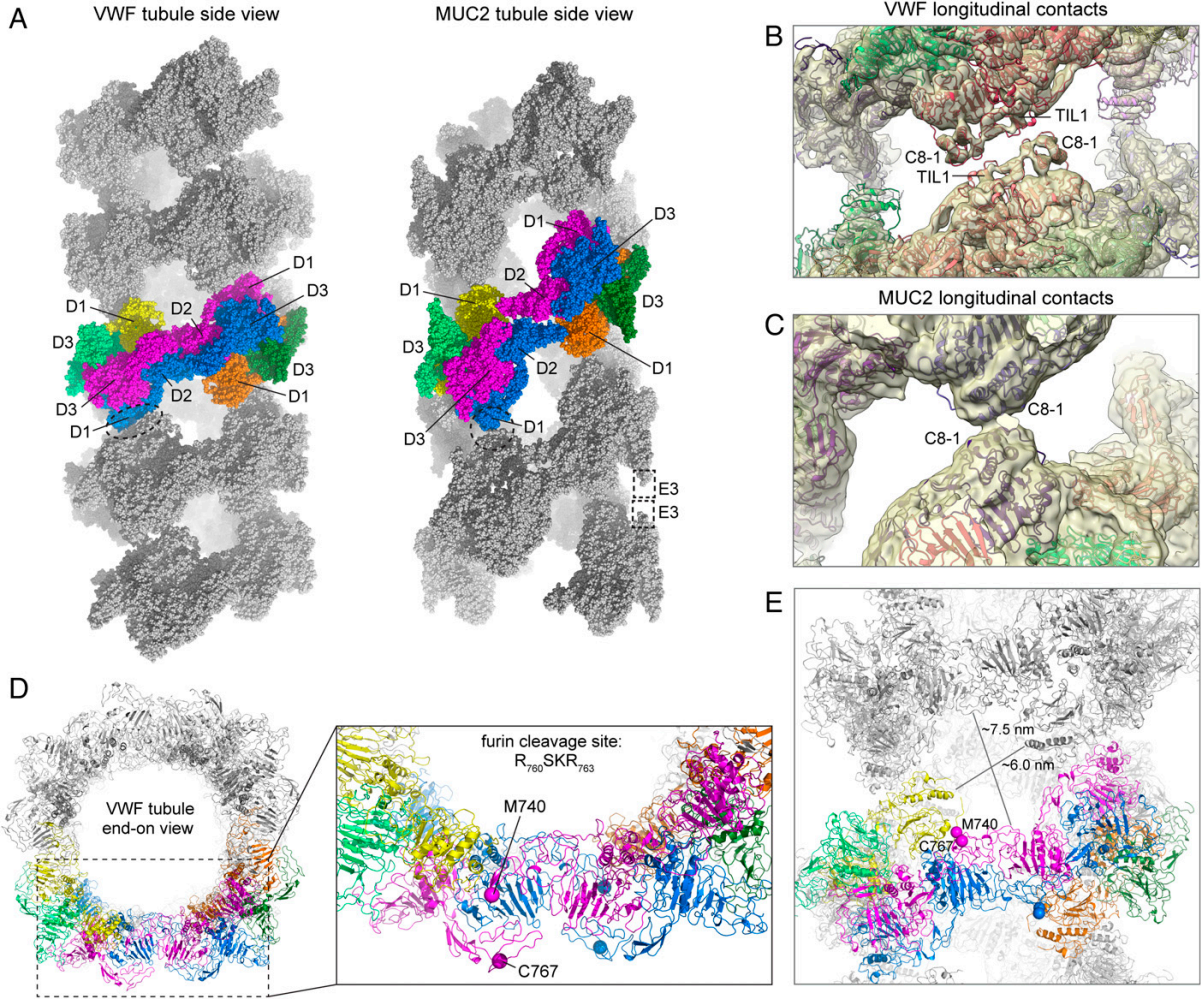
Tubule models for VWF and MUC2. (*A*) Tubules were generated for VWF and MUC2 using the two-bead models. Space-filling sphere representations are shown with two beads from each tubule colored according to Fig. 3*B*. Positions of the D assemblies are indicated. Points of contact between beads in successive helical turns are in a dashed oval and circle. The apparent contact between E3 modules the MUC2 tubule (corresponding to the gray triangle in Fig. 2*F*) is represented by dashed rectangles. This contact could not be explicitly modeled because the map was poor in this region. (*B*) The VWF tubule model is shown within the map from the helical reconstruction with imposed D1 symmetry. Domains making contacts between helical turns (longitudinal contacts) are labeled. (*C*) The MUC2 tubule model is shown within the map from the helical reconstruction with imposed D2 symmetry. (*D*) The VWF tubule is presented end-on, with the polypeptide chain in cartoon and colored as in panel *A*. The boxed region is expanded to the *Right*. The endpoints of the chains that could be modeled in the VWF two-bead map are shown as large spheres and labeled. (*E*) The flexible polypeptide chain in the vicinity of the furin cleavage site is exposed to the holes, or windows, in the tubule seen from the side, as in panel *A*. Approximate dimensions of the windows are indicated.

Using the bead models and the known rotation and translation between beads, full tubules of each protein were generated and validated by comparison with the helical reconstruction maps. As anticipated from the helical reconstructions (Fig. 2 *E* and *F*, black arrows), successive turns make only minimal contacts in the tubule models (Fig. 4*A* and Movie S2). In the VWF tubule, reciprocal contacts are made between C8-1 and TIL1 modules of interacting beads (Fig. 4*B*), with a total interface area of about 500 Å^2^. His288, which could make pH-sensitive interactions, is at this interface, but this histidine is not highly conserved in VWF (41). In the MUC2 tubule, C8-1 modules from each bead contact one another (Fig. 4*C*), with an interface area of less than 200 Å^2^. The MUC2 tubule also has a second contact between helical turns (Fig. 2 *F*, gray arrows). Although the resolution of the map was poor at this second contact, the docked D3 assembly crystal structure (42) suggested that it is formed by the carboxyl-terminal region of the E3 module or by the His_6_ tag present in the recombinant protein following E3 (Fig. 4*A*). Importantly, the His_6_ tag was not essential for tubule formation, as the D1D2D3 fragment produced with an amino-terminal His_6_ tag that was removed proteolytically during purification retained the ability to form tubules (*SI Appendix*, Fig. S4).

A benefit of the VWF tubule model is that it enables a consideration of furin cleavage in the context of the helical assembly. As noted above, the region surrounding the furin cleavage site was disordered and could not be modeled, but its junctions with the rest of the protein clearly indicate that the cleavage site is exposed in the “window” of the VWF tubule, which has dimensions of about 7 × 6 nm (Fig. 4 *D* and *E*). The cleavage site is also likely to be accessible from the tubule exterior. For comparison, crystal structures of furin (e.g., PDB code 5MIM) reveal that it is about 6 × 5 × 4 nm. Furthermore, only the furin active site would need to reach the cleavage site in the VWF tubule. Thus, the structure of the VWF tubule is consistent with furin cleavage occurring after tubule assembly in vivo (27).

## Discussion

The results reported here show that tubule formation is a property not only of VWF but also of the MUC2 segment homologous to the VWF tubule core. Since intact mucins are not known to form tubules in vivo, there are two possible explanations for in vitro tubule formation by the MUC2 amino terminus. One explanation is that a propensity for tubule formation is intrinsic to the structural organization of the VWF/mucin amino terminus but is generally suppressed in full-length mucins. The other is that some full-length mucins also form, or once formed, tubules in nature and that this ability was under positive functional selection prior to the evolutionary branching of VWF from mucins. Regardless of whether tubule formation is a structural proclivity or a product of evolutionary pressure, it should be noted that the observed assembly of the MUC2 segment into two-start helices and the VWF segment into one-start helices is not a fundamental difference. Although one-start and two-start helices are discrete structural states, they can be quite similar in the lateral packing of subunits along the helix path and in the distances and interactions between neighboring rungs. Consequently, even a single point mutation in a large helical assembly can favor a switch between one- and two-start helices (43).

To discuss the possibility that the tendency to form tubules is inherent to the symmetry and physical properties of the mucin amino terminus but is suppressed in intact mucins, we must first explain why such a tendency should exist. Then, we may consider both why and how mucins might avoid assembling as tubules. Protein quaternary structures, particularly those with dihedral symmetry, are prone to supramolecular assembly (44). Self-interacting surfaces appear in multiple copies in high-symmetry quaternary structures, enabling them to engage in cooperative interactions. The symmetry of the mucin/VWF bead and the high effective concentration of beads in a filament permit multiple small surface patches, each individually making weak interactions, to cooperate in stabilizing the helical tubule.

The explanation for why mucins nevertheless do not appear to form tubules in vivo is likely to lie in mucin segments that differ from VWF. Gel-forming mucins are dominated by the central PTS region (Fig. 1*A*), which is thousands of amino acid residues long and bears a huge additional mass of carbohydrates (45). Accommodating this amount of material around a compact, tubular scaffold is likely to be difficult due to the entropic cost of restricting the volume available for PTS polymer dynamics (see calculations in *Materials and Methods*). Considering tubules and filaments in the framework of polymer brushes, filaments have a much lower density of PTS regions along their lengths. Specifically, the degenerate helix of the MUC2 filament spans 50 nm per turn (rotation of 98.5° and translation of 13.7 nm per bead), such that about 3.6 beads, or 7.2 PTS regions, are present per turn. In 50 nm of a MUC2 helical tubule, 14.4 beads or 28.8 PTS regions would be present, i.e., the MUC2 tubule has four times the “grafting density” of the filament brush along its length (46). Although the tubule does displace the grafting sites about 12.5 nm off the helix axis, the resulting minor compensation in grafting density does not outweigh the massively increased number of polymers per unit length of scaffold.

If PTS regions are sufficient to antagonize tubule formation due to the steric and entropic argument above, this concept would apply to all gel-forming mucins. However, some mucins may also use CysD domains to stabilize an extended filamentous structure. Human MUC2 contains two CysD domains near the beginning of the PTS region (Fig. 1*A*), whereas MUC5B and MUC5AC contain seven and nine CysD domains, respectively. In the structure of the MUC2 filament (22), the CysD1 domain, at the end of the amino-terminal region (Fig. 1*B*), projects outward from one bead, with the aid of an intervening ∼40-amino acid, threonine-rich O-glycosylated segment, and docks onto the neighboring bead. This interaction is likely to help fix the relative orientation of adjacent beads in the filament. Interestingly, the CysD1 contact is a ternary interaction involving both molecules of the neighboring bead and is thus comparable to the ternary interaction involving the bridge of VWF, which appears to help fix the relative orientation of successive beads in the VWF tubule (Fig. 3*C*). CysD domains may have evolved to stabilize extended, filamentous forms of mucin assembly intermediates and then were perhaps duplicated to engage in other protein–protein interactions during mucin assembly or extracellular function (6, 47–49).

While there is no evidence as yet of mucins naturally forming tubules, VWF has clearly evolved to utilize tubules for functional purposes. The residue-resolution structure of VWF, presented here, enabled an analysis of the molecular features that produce the tight, one-start helix previously visualized only at low resolution (21) or by homology modeling (22). An important lesson from the current analysis is that the different manners in which the mucin and VWF amino termini self-assemble, i.e., filaments vs. tubules, are not obvious from inspecting their sequences. The different supramolecular structures likely stem from multiple, subtle, and distributed amino acid sequence features of the two glycoproteins. Indeed, sequence alignments of the MUC2 and VWF amino-terminal regions show remarkable conservation for such functionally different molecules (22, 40, 50). The major deviations, aside from the furin cleavage site, which exists only in VWF, are in loops in the D1 assemblies. These loops point into the center of the VWF tubule and appear to have diverged from mucins because they are weakly constrained, not as a result of selection to support tubule formation. Instead, the key VWF amino acid residues that make contacts stabilizing the helical configuration of beads, such as shown in Fig. 3*C*, are within well-aligned sequence regions. Although many of these key amino acids are hydrophobic in both VWF and MUC2, the end of the TIL2 module and the E2 module of VWF have an increased content of aromatic amino acids compared to the homologous region of MUC2 (nine phenylalanines and tyrosines in VWF vs. four in MUC2). The aromatic residues likely rigidify these modules. Correspondingly, basic residues arose at appropriate positions distributed in various loops of D1 and D2 of VWF to complete the cation–π interactions described. The small number of differences in the bridge region and its contacts, compared to the large bead structure, shows how readily supramolecular assemblies can arise and become stabilized in the context of evolutionary adaptations.

## Materials and Methods

### Protein Production and Purification.

The plasmid for producing MUC2 D1D2D3 (residues 21 to 1259 followed by a His_6_ tag) was described previously (42). MUC2 D1D2D3, with an amino-terminal His_6_ tag and TEV protease cleavage site and lacking a carboxy-terminal His_6_, was produced by restriction-free cloning. VWF gene fragments were amplified from clone 100068741 of hORFeome5.1 with primers containing an amino-terminal AflII site and a carboxy-terminal His_6_ tag coding sequence plus an EcoRI site. VWF D1D2 (residues 1 to 745) and VWF D1D2D3 (residues 1 to 1241) were inserted into the pCDNA3.1 vector. Plasmids were propagated in and purified from *Escherichia coli* XL-1 cells. Proteins were produced by transient transfection of these plasmids into HEK 293F cells using the polyethylenimine (PEI) Max reagent (Polysciences, Inc.) with a 1:3 ratio (wt/wt) of DNA to PEI at a concentration of 1 million cells per milliliter. Cells were maintained in FreeStyle 293 medium. Six days after transfection, the culture medium was collected and centrifuged for 15 min at 500 g to pellet cells. The supernatant was then centrifuged for 15 min at 3,000 g to pellet any remaining particulate matter. The supernatant from this second centrifugation was filtered through a 0.45-μm filter, and the His_6_-tagged proteins were purified by nickel-nitrilotriacetic acid (Ni-NTA) chromatography. MUC2 proteins were buffer exchanged into 10 mM Tris (pH 7.5) and 50 mM NaCl and concentrated to 3 mg/mL. VWF proteins were concentrated to 0.6 mg/mL and dialyzed against 20 mM 4-morpholineethanesulfonic acid (MES; pH 5.6), 10 mM CaCl_2_, and 150 mM NaCl. Mucins and VWF have calcium-binding sites in their VWD domains (32, 37), which appear to be loaded with calcium in the purified proteins. Nevertheless, the addition of calcium was not deleterious to higher order assembly, so it was supplied.

### Dynamic Light Scattering.

Prewarmed samples at 37 °C were placed in a clear-bottom black 384-well plate and diluted to a final concentration of 0.3 mg/mL in 50 mM MES buffer, at varying pH values, and 150 mM NaCl. All samples and buffers were passed through 0.1-µm filters. DLS data were recorded using a Dynapro Plate Reader III (Wyatt Technology) prewarmed to 37 °C. Data were processed with the supplied DYNAMICS software.

### Sample Preparation for EM.

For negative staining, purified MUC2 D1D2D3 was diluted to 0.3 mg/mL in 50 mM MES (pH 5.4), 225 mM NaCl, and 10 mM CaCl_2_ and incubated at 37 °C for 24 h. Dialyzed VWF proteins were diluted to 0.3 mg/mL in 50 mM MES (pH 6.0), 150 mM NaCl, and 10 mM CaCl_2_ at a 2:1 molar ratio of VWF D1D2 to VWF D3 and incubated at 37 °C for 24 h. After incubation, the proteins were diluted in their respective buffers to a concentration of 0.03 mg/mL, and 3 µL was applied to 60-s glow discharged (PELCO easiGlow) carbon-coated 300 mesh copper grids (Electron Microscopy Sciences). Grids were stained with a 2% uranyl acetate and allowed to dry. Samples were visualized using a Tecnai T12 electron microscope (Thermo Fisher Scientific) equipped with a OneView camera (Gatan). For cryo-EM, 3 µL of the incubated proteins was applied to 90-s glow discharged Quantifoil R 1/2, 300 mesh copper grids. Using a Vitrobot Mark IV plunger (Thermo Fisher Scientific), grids were plunge frozen from a chamber held at 10 °C and 100% humidity into liquid ethane cooled by liquid nitrogen.

### Cryo-EM Image Acquisition.

Cryo-EM data were collected on a Titan Krios G3i transmission electron microscope (Thermo Fisher Scientific) operated at 300 kV. Movies were recorded on a K3 direct detector (Gatan) installed behind a BioQuantum energy filter (Gatan) using a slit of 20 eV. Movies were recorded in counting mode at a nominal magnification of 105,000×, corresponding to a physical pixel size of 0.83 Å. The dose rate was set to 23 e-/pixel/s, and the total exposure time was 1.5 s, resulting in an accumulated dose of ∼48 e-/Å^2^. Each movie was split into 45 frames of 0.033 s. Nominal defocus range was −1 to −2 μm. EPU (Thermofisher) was used for automated data collection, in which a single image was collected from the center of each hole. Image shift was used to navigate within 10-µm arrays and stage shift to move between arrays.

### Cryo-EM Image Processing and Atomic Model Fitting.

Recorded movies were imported into CryoSPARC v 3.2 (51) and subjected to Patch-Based motion correction and Patch-Based contrast transfer function (CTF) estimation. Micrographs with better than a 4.0-Å CTF fit resolution were retained for further processing. For MUC2, this resulted in 2,578 micrographs retained from an initial 3,553, while for VWF, this resulted in 4,996 retained from an initial 6,297. Particles were picked using the filament tracer with a separation distance between boxes of 70 Å for MUC2 and 60 Å for VWF. Initial numbers of overlapping particles were 957,320 and 552,573 for MUC2 and VWF, respectively. Extensive two-dimensional classification yielded 515,422 and 88,715 particles. These particles were used for ab initio helical refinement performed with constraints on in-plane rotation and longitudinal shifts but no additional symmetry, starting with a cylindrical model of 110-Å inner diameter and 270-Å outer diameter. This step produced an initial 3D map and initial helical parameters. The generated 3D helical volumes and parameters were used for further helical refinement of the same particles with imposed D2 and two-start symmetry for MUC2 and D1 for VWF, while iteratively refining helical parameters. Particles were symmetry expanded with the resulting helical parameters, and local refinement was performed without symmetry using a mask encompassing two successive beads along the helical path. A 20° limit was placed on the rotation search extent in the local refinement to prevent overfitting. Maps were density modified with default parameters and used for model building (52). Alternative masks, encompassing two beads interacting longitudinally along the tubule long axes, did not produce maps of higher resolution.

### Model Building, Refinement, and Analysis.

The previously reported MUC2 D1D2D3CysD1 bead structure encompassing amino acids 27 to 1197 (PDB ID 6TM2) was docked into the MUC2 D1D2D3 tubule structure using Chimera’s Fit in Map tool (53). The MUC2 D3 dimer (PDB ID 6RBF) was aligned to the docked bead, extending the model to residue 1247. There was missing density in the bridge region between assemblies D2 and D3, and the residues without clear density were removed from the structure. Map local resolution figures were generated using ChimeraX (54) from CryoSPARC's local resolution estimation, and model figures were generated using Pymol (55).

A VWF bead model was generated using SWISS-MODEL (56) based on the MUC2 D1D2D3CysD1 bead structure. This model was docked into the VWF D1D2D3 tubule map, along with the previously reported monomeric VWF D'D3 structures (PDB IDs 6N29, 7KWO). A hybrid structure constructed from elements of these two docked models was rebuilt and refined by iterative cycles of Phenix (57) real-space refinement and interactive rebuilding in Coot. The final model spanned amino acid residues 31 to 1207 with gaps in the regions 211 to 220 and 740 to 767 due to missing density.

### Calculations of the Partial Specific Volume of the MUC2 PTS Region.

To obtain the spatial requirements of a PTS region, we calculated its approximate partial specific volume (*v*), i.e., the change in volume of solution when the PTS region is added. The protein mass of MUC2 residues 1398 to 4331 (the PTS region containing the CysD2 domain) is about 295,000 g/mol. Using the *v* for proteins of 0.73 cm^3^/g, the protein component of the PTS region would occupy about 360 nm^3^/molecule. It has been claimed that mucins can be 80 or 90% carbohydrate by weight (45, 58). Taking a conservative estimate that the carbohydrate mass of the PTS region is 4 times the protein mass, and using a value for *v* of 0.61 cm^3^/g for carbohydrates, the carbohydrate component would occupy about 1,200 nm^3^/molecule. The total *v* would thus be about 1,560 nm^3^ per PTS region. As two PTS regions are associated with each bead, 3,120 nm^3^ would be required simply for the PTS mass per bead. This volume corresponds to a sphere with a diameter of about 18 nm, which is larger than the 15 nm available per bead along the tubule. The solvated PTS region would occupy a much larger volume.

## Supplementary Material

Supplementary File

Supplementary File

Supplementary File

## Data Availability

The atomic coordinates have been deposited in the Protein Data Bank with the codes 7PP6 (MUC2 beads), 7POV (MUC2 tubules), 7PMV (VWF beads), and 7PNF (VWF tubules). Cryo-EM density maps have been deposited in the Electron Microscopy Data Bank with accession codes 13580 (MUC2 beads, local reconstruction), 13575 (MUC2 helical reconstruction), 13541 (VWF beads, local reconstruction), and 13547 (VWF helical reconstruction).
