# Supplementary material for: Helical self-assembly of a mucin segment suggests an evolutionary origin for von Willebrand factor tubules
Source: Proc Natl Acad Sci U S A. 2022 Apr 4;119(15):e2116790119. doi: 10.1073/pnas.2116790119 (PMC9169620; doi:10.1073/pnas.2116790119)
Supplement: Supplementary File [file pnas.2116790119.sapp.pdf]

PNAS
www.pnas.org

Supplementary Information for

Helical Self-assembly of a Mucin Segment Suggests an Evolutionary Origin for von Willebrand Factor Tubules

Gabriel Javitt*, Deborah Fass*

*Corresponding authors:

Email: gabriel.javitt@Weizmann.ac.il, deborah.fass@weizmann.ac.il

This PDF file includes:

Figures S1 to S4
Table S1
Legends for Movies S1 to S2

Other supplementary materials for this manuscript include the following:

Movies S1 to S2

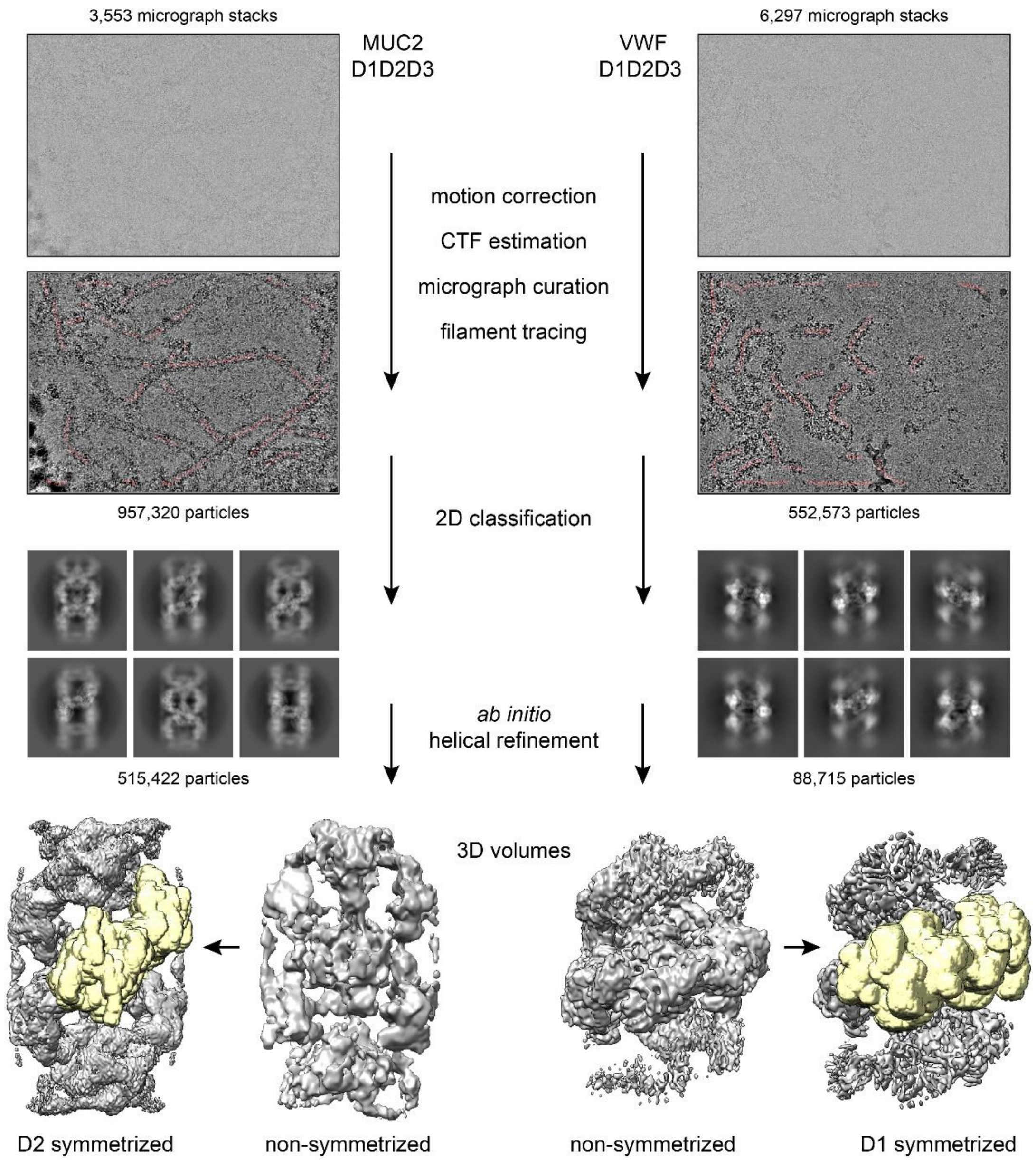

Figure S1. Cryo-EM workflow for particle dataset generation. Micrographs were subjected to motion correction, CTF estimation, and curated to only those with better than a 4.0 Å CTF fit resolution. Particles were picked using the filament tracer feature and aligned into 2D classes. Junk particles were discarded, and particles were reconstructed with helical refinement using a featureless cylinder as a starting model. From the initial models, dyad symmetry for both VWF and MUC2, and two-start symmetry for MUC2, were apparent and used for subsequent helical refinement as reported in the main text. Masks used for local refinement are shown in yellow.

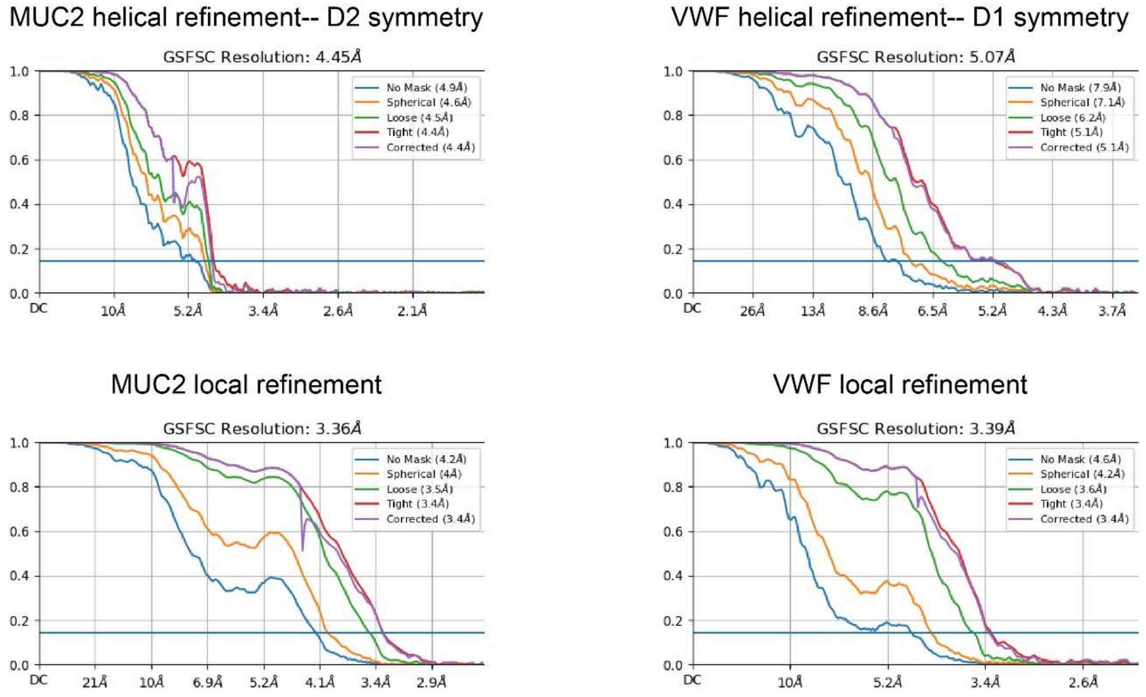

Figure S2. Fourier shell correlation (FSC) curves. Resolution at a FSC cut-off of 0.143 is shown above each plot.

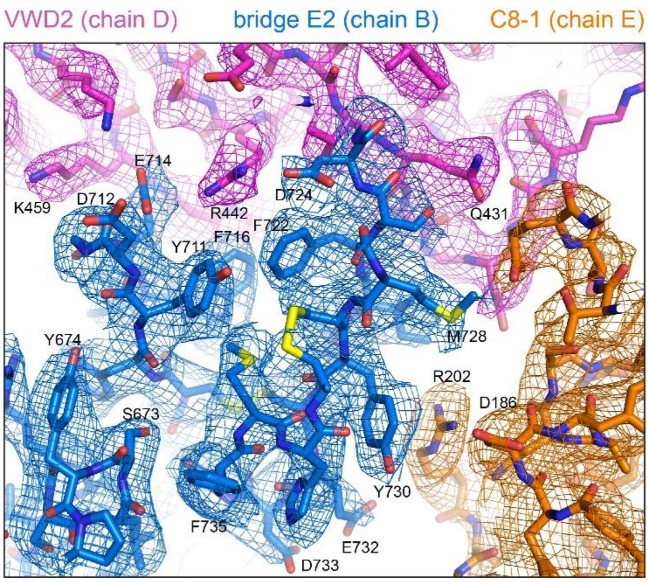

Figure S3. Map density is shown in the region of the ternary interaction presented in Figure 3*C*. The map is shown within 1.6 Å of atoms in the model.

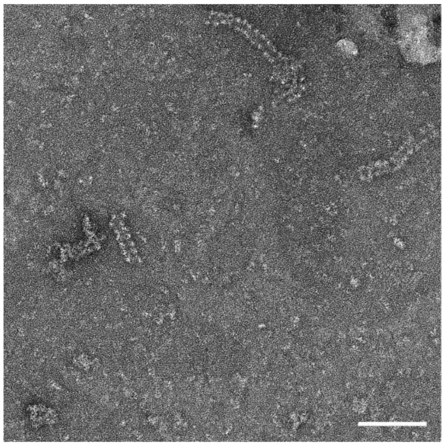

Figure S4. Tubules of MUC2 D1D2D3 lacking a carboxy-terminal His_6 tag. This version of the protein was produced with a His_6 tag and TEV cleavage site following the signal sequence at the amino terminus of the protein. The His_6 tag was removed by TEV cleavage, and the resulting protein, which did not bind a Ni-NTA column, was incubated in 50 mM MES, pH 5.4, 225 mM NaCl, 10 mM $CaCl_2$ at 37°C for 24 hr. Three microliters of 0.03 mg/ml solution were applied to carbon-coated grids and imaged by negative stain TEM. The scale bar is 100 nm.

Table S1. Cryo-EM data collection, refinement, and validation statistics

	#1 VWF D1D3D'D3 (EMDB-13541) (PDB 7PMV)	#2 MUC2 D1D2D3 (EMDB-13580) (PDB 7PP6)
**Data collection and processing**		
Microscope	Titan Krios	Titan Krios
Magnification	105,000x	105,000x
Voltage (kV)	300	300
Detector	BioQuantum-K3	BioQuantum-K3
Data collection software	EPU	EPU
Electron exposure (e–/Å^2)	48	48
Defocus range (μm)	0.5-2.0	0.5-2.0
Pixel size (Å)	0.83	0.83
**Data processing**		
Number of micrographs	4996	2578
Symmetry imposed	D1	D2
Initial particle images (no.)	552,573	957,320
Final particle images (no.)	88,715	515,422
Map resolution (Å)		
FSC threshold 0.143	3.4	3.4
Map resolution range (Å)	2.8-6.0	2.8-7.3
**Refinement**		
Initial model used (PDB code)	6N29 7KWO	6TM2 6RBF
Model resolution (Å)		
FSC threshold 0.143	3.26	3.35
Map sharpening *B* factor (Å^2)	77.8	90.6
Model composition		
Non-hydrogen atoms	35347	34860
Protein residues	4560	4592
Ligands	39	28
B factors (Å^2) mean		
Protein	45.33	43.42
Ligand	61.00	80.20
R.m.s. deviations		
Bond lengths (Å)	0.009	0.010
Bond angles (°)	1.271	1.484
Validation		
MolProbity score	2.31	2.19
Clash score	11.88	5.07
Poor rotamers (%)	0.1	2.5
Ramachandran plot		
Favored (%)	81.15	86.93
Allowed (%)	18.52	12.72
Disallowed (%)	0.33	0.35
Cβ outliers (%)	0.00	0.35
Peptide plane (%)		
Cis proline/general	4.2/0.0	5.6/0.0
Twisted proline/general	0.0/0.1	1.9/0.1
CaBLAM outliers (%)	10.66	7.86

Movie S1 (separate file). Three MUC2 beads are shown in cartoon representation with each polypeptide colored from blue (amino terminus) to red (carboxy terminus). The movie starts with the beads in the configuration of the MUC2 filament and morphs to the configuration of the MUC2 tubule.

Movie S2 (separate file). The VWF tubule, the MUC2 tubule, and the MUC2 filament (containing the CysD1 domain) are shown rotating around their long axes. Different protein chains in the central two beads are distinguished by color. Blue and magenta are complete D1D2D3 segments.
